# Use of multiple polygenic risk scores for distinguishing schizophrenia-spectrum disorder and affective psychosis categories in a first-episode sample; the EU-GEI study

**DOI:** 10.1017/S0033291721005456

**Published:** 2023-06

**Authors:** Victoria Rodriguez, Luis Alameda, Diego Quattrone, Giada Tripoli, Charlotte Gayer-Anderson, Edoardo Spinazzola, Giulia Trotta, Hannah E. Jongsma, Simona Stilo, Caterina La Cascia, Laura Ferraro, Daniele La Barbera, Antonio Lasalvia, Sarah Tosato, Ilaria Tarricone, Elena Bonora, Stéphane Jamain, Jean-Paul Selten, Eva Velthorst, Lieuwe de Haan, Pierre-Michel Llorca, Manuel Arrojo, Julio Bobes, Miguel Bernardo, Celso Arango, James Kirkbride, Peter B. Jones, Bart P. Rutten, Alexander Richards, Pak C. Sham, Michael O'Donovan, Jim Van Os, Craig Morgan, Marta Di Forti, Robin M. Murray, Evangelos Vassos

**Affiliations:** 1 Psychosis Studies, Institute of Psychiatry, Psychology and Neuroscience, King's College of London, London, UK; 2 Instituto de Investigación Sanitaria de Sevilla, IBiS, Hospital Universitario Virgen del Rocío, Department of Psychiatry, Universidad de Sevilla, Sevilla, Spain; 3 Service of General Psychiatry, Treatment and Early Intervention in Psychosis Program, Lausanne University Hospital (CHUV), Lausanne, Switzerland; 4 Social, Genetics and Developmental Psychiatry Centre, Institute of Psychiatry, Psychology and Neuroscience, King's College London, London, UK; 5 Department of Health Service and Population Research, Institute of Psychiatry, Psychology and Neuroscience, King's College London, London, UK; 6 Psychiatry Residency Training Program, Faculty of Medicine and Psychology, Sapienza University of Rome, Rome, Italy; 7 Department of Experimental Biomedicine and Clinical Neuroscience, University of Palermo, Palermo, Italy; 8 Psylife Group, Division of Psychiatry, University College London, London, UK; 9 Department of Mental Health and Addiction Services, ASP Crotone, Crotone, Italy; 10 Section of Psychiatry, Department of Biomedicine, Neuroscience and advanced Diagnostic (BiND), University of Palermo, Palermo, Italy; 11 Section of Psychiatry, Department of Neuroscience, Biomedicine and Movement, University of Verona, Verona, Italy; 12 Bologna Transcultural Psychosomatic Team (BoTPT), Department of Medical and Surgical Science, Alma Mater Studiorum Università di Bologna, Bologna, Italy; 13 Neuropsychiatrie Translationnelle, INSERM, U955, Faculté de Santé, Université Paris Est, Créteil, France; 14 Rivierduinen Institute for Mental Health Care, Leiden, The Netherlands; 15 Department of Psychiatry and Neuropsychology, School for Mental Health and Neuroscience, South Limburg Mental Health Research and Teaching Network, Maastricht University Medical Centre, Maastricht, The Netherlands; 16 Department of Psychiatry, Early Psychosis Section, Amsterdam UMC, University of Amsterdam, Amsterdam, The Netherlands; 17 Department of Psychiatry, Icahn School of Medicine at Mount Sinai, New York, USA; 18 Université Clermont Auvergne, Clermont-Ferrand, France; 19 Department of Psychiatry, Psychiatric Genetic Group, Instituto de Investigación Sanitaria de Santiago de Compostela, Complejo Hospitalario Universitario de Santiago de Compostela, Santiago, Spain; 20 Instituto de Investigación Sanitaria del Principado de Asturias (ISPA), Oviedo, Spain; 21 Department of Medicine, Psychiatry Area, School of Medicine, Universidad de Oviedo, Centro de Investigación Biomédica en Red de Salud Mental (CIBERSAM), Oviedo, Spain; 22 Barcelona Clinic Schizophrenia Unit, Neuroscience Institute, Hospital Clinic of Barcelona, University of Barcelona, Institut d'Investigacions Biomèdiques August Pi I Sunyer, Biomedical Research Networking Centre in Mental Health (CIBERSAM), Barcelona, Spain; 23 Department of Child and Adolescent Psychiatry, Institute of Psychiatry and Mental Health, Hospital General Universitario Gregorio Marañón, School of Medicine, Universidad Complutense, IiSGM, CIBERSAM, Madrid, Spain; 24 Department of Psychiatry, University of Cambridge, Cambridge, UK; 25 CAMEO Early Intervention Service, Cambridgeshire & Peterborough NHS Foundation Trust, Cambridge, UK; 26 Division of Psychological Medicine and Clinical Neurosciences, MRC Centre for Neuropsychiatric Genetics and Genomics, Cardiff University, Cardiff, UK; 27 Centre for Genomic Sciences, Li KaShing Faculty of Medicine, The University of Hong Kong, Hong Kong, Hong Kong; 28 Department Psychiatry, Brain Centre Rudolf Magnus, Utrecht University Medical Centre, Utrecht, The Netherlands

**Keywords:** Affective psychosis, bipolar disorder, diagnosis, genetics, polygenic score, psychosis, psychotic depression, schizophrenia-spectrum disorder

## Abstract

**Background:**

Schizophrenia (SZ), bipolar disorder (BD) and depression (D) run in families. This susceptibility is partly due to hundreds or thousands of common genetic variants, each conferring a fractional risk. The cumulative effects of the associated variants can be summarised as a polygenic risk score (PRS). Using data from the EUropean Network of national schizophrenia networks studying Gene-Environment Interactions (EU-GEI) first episode case–control study, we aimed to test whether PRSs for three major psychiatric disorders (SZ, BD, D) and for intelligent quotient (IQ) as a neurodevelopmental proxy, can discriminate affective psychosis (AP) from schizophrenia-spectrum disorder (SSD).

**Methods:**

Participants (842 cases, 1284 controls) from 16 European EU-GEI sites were successfully genotyped following standard quality control procedures. The sample was stratified based on genomic ancestry and analyses were done only on the subsample representing the European population (573 cases, 1005 controls). Using PRS for SZ, BD, D, and IQ built from the latest available summary statistics, we performed simple or multinomial logistic regression models adjusted for 10 principal components for the different clinical comparisons.

**Results:**

In case–control comparisons PRS-SZ, PRS-BD and PRS-D distributed differentially across psychotic subcategories. In case–case comparisons, both PRS-SZ [odds ratio (OR) = 0.7, 95% confidence interval (CI) 0.54–0.92] and PRS-D (OR = 1.31, 95% CI 1.06–1.61) differentiated AP from SSD; and within AP categories, only PRS-SZ differentiated BD from psychotic depression (OR = 2.14, 95% CI 1.23–3.74).

**Conclusions:**

Combining PRS for severe psychiatric disorders in prediction models for psychosis phenotypes can increase discriminative ability and improve our understanding of these phenotypes. Our results point towards the potential usefulness of PRSs in specific populations such as high-risk or early psychosis phases.

## Introduction

More than 100 years have passed since Kraepelin established the dichotomy of manic-depression and dementia praecox as the two fundamental pillars of psychotic illness, which still constitutes the basis of current diagnostic criteria (Kraepelin, 1899). However, it is a matter of debate whether schizophrenia (SZ) and bipolar disorder (BD) are discrete illnesses or conditions which are part of an overall conceptual continuum (Craddock & Owen, 2010; Demjaha, MacCabe, & Murray, 2012; Murray et al., 2004). Given the high heritability of these disorders (Smoller et al., 2019), genetic tools can be used to dissect possible biological differences between these diagnostic categories.

Genome-Wide Association Studies (GWAS) have shown that, as with other psychiatric conditions, many hundreds or thousands of common alleles influence susceptibility to SZ and BD (Ripke et al., 2013; Stahl et al., 2019). We can calculate individual polygenic risk scores (PRS) based on the summation of the carried risk of single nucleotide polymorphisms (SNPs) selected in a discovery GWAS according to their *p*-value, weighted by their effect size (Dudbridge, 2013; Purcell et al., 2009). GWAS analyses of case–control samples by the Psychiatric Genomics Consortium (PGC) have estimated SNP-heritability for SZ, BD and Major Depressive Disorder (MDD) as about 22.2% (Ripke, Neale, Corvin, & Walters, 2014), 18.2% (Stahl et al., 2019), and 8.5% (Wray et al., 2018) respectively.

In line with the previous family and twin studies (Cardno & Owen, 2014; Cardno, Rijsdijk, Sham, Murray, & McGuffin, 2002; Craddock & Owen, 2005), GWAS findings have also supported the notion of genetic overlap among severe mental disorders. A study from the Cross-Disorder Group of PGC (Lee et al., 2019) showed genetic correlation using common SNPs, of around 0.70 between SZ and BD, 0.34 between SZ and MDD, and 0.36 between BD and MDD.

On the other hand, some studies provide support for a link between genetic predisposition and current diagnostic categories. A study investigating diagnostic subcategories across the psychosis spectrum employing PRS-SZ and PRS-BD (Tesli et al., 2014) provided some validation for the existence of subcategories across the SZ and BD continuum. In line with this, in a more recent study, PRS for SZ discriminated SZ from BD; and within BD cases, between those with and without psychosis (Allardyce et al., 2017). Moreover, Markota et al. (Markota et al. 2018), found that PRS-SZ seemed to be more closely related with bipolar disorder type I (BD-I) with psychotic symptoms during manic phases as compared with BD-I with psychotic symptoms during depressive episodes or those without psychosis. Taken together, these findings shed light on the genetic architecture of these severe mental disorders and support the discriminability potential of the polygenic score on diagnostic categories.

To the best of our knowledge, only one study has previously examined the relationship between different diagnostic categories by employing three polygenic scores, specifically PRS-SZ, PRS-BD and PRS-MDD (Charney et al., 2017), but only examined cases within the BD spectrum. They found a PRS-SZ gradient among affective psychotic categories, with the highest association being schizoaffective followed by BD-I and BD type II (BD-II).

Consistent evidence suggests that cognitive deficits can be considered a core feature for SZ (Green, 2006). It has been long accepted that subjects affected by SZ perform worse than those with BD on a variety of cognitive domains (Goldberg, 1999; Zanelli et al., 2010), and this has been validated by a meta-analysis showing that subjects with BD show better cognitive performance than those with SZ (Krabbendam, Arts, van Os, & Aleman, 2005). Although there remains debate over the extent to which these differences in cognition predate or follow the onset of psychosis (Trotta, Murray, & Maccabe, 2015), it is important to include genetic differences in cognitive ability and intelligence in models aiming to differentiate subgroups of patients with psychosis.

Given the above, this study aims to explore the potential of joint modelling PRS from three major mental disorders (SZ, BD, D) and intelligence quotient (IQ) for discriminating affective psychosis (AP) from schizophrenia-spectrum disorder (SSD). We built on a previous study from South London, where we have shown that PRS-SZ differentiated SZ from other psychoses (Vassos et al., 2017).

## Methods

### Sample

The present study is based on the case–control sample from the (EUropean Network of national schizophrenia networks studying Gene-Environment Interactions) EU-GEI study; a multisite incidence and case–control study of genetic and environmental determinants involved in the development of psychotic disorders(Gayer-Anderson et al., 2020).

The baseline sample comprises a total of 2627 participants, including 1130 patients aged 18 to 64 years who were resident within the study areas and presented to the adult psychiatric services between 1 May 2010 and 1 April 2015 in 17 sites across 6 countries: England, the Netherlands, Italy, France, Spain and Brazil. All participants provided informed, written consent. Ethical approval was provided by relevant research ethics committees in each of the study sites. All data were stored anonymously.

Cases were selected if they were experiencing their first episode of psychosis (FEP) including SZ and related psychosis, BD and Major Depression Disorder with Psychotic features (MDD-P). In addition, 1497 unaffected screened controls with no lifetime psychotic disorder were also recruited in the areas served by the services with a quota sampling approach, a non-probability sampling method in which a specific subgroup is chosen in order to represent the local population. Details on recruitment of the sample are provided in online Supplementary Material; and further information about the methodology of the study is available on the EU-GEI website (http://www.eu-gei.eu/) and can be found in previous publications (Di Forti et al., 2019; Gayer-Anderson et al., 2020; Jongsma, Gayer-Anderson, Lasalvia, Quattrone, & Mulè, 2018; Quattrone et al., 2018).

One of the problems when using current PRS is the limited predictive power in multi-ethnic samples as they have derived from mostly European samples (Curtis, 2018). This has been shown in a previous study on FEP patients (Vassos et al., 2017), where PRS-SZ had much lower predictive power in the African ancestry population. Given the wide variance across ancestral groups, for the scope of the present study, we constrained the sample to those categorised as of European ancestry based on PCA (details provided in online Supplementary Material). Characteristics of the final sample are summarised in Table 1.

**Table 1.** Sociodemographic of European subsample (*n* = 1659), case–control comparisons

Descriptive at baseline	Number (%)/Mean (s.d.)		Statistics		Number (%)/Mean (s.d.)	Statistics	
Control *n* = 1005	Schizophrenia-spectrum disorder *n* = 409	Tests (df)	*p* value	Affective psychosis *n* = 164	Tests (df)	*p* value	
Gender			*Χ*^2^ (1) = 50.54	<0.001		*Χ*^2^ (1) = 0.67	0.413
Male	474 (47.2)	278 (68)			83 (50.6)		
Female	531 (52.8)	131 (32)			81 (49.4)		
Age (years)	36.9 (13)	31.63 (10.92)	*Z* = 7.21	<0.001	32.84 (11.56)	*Z* = −3.76	<0.001
Ever used Cannabis			*Χ*^2^ (1) = 40.26	<0.001		*Χ*^2^ (1) = 15.9	<0.001
No	528 (53)	136 (34.2)			58 (36)		
Yes	469 (47)	262 (65.8)		<0.001	103 (64)		
Education level			*Χ*^2^ (2) = 81.22				
No qualification	40 (4)	65 (16.1)			25 (15.3)	*Χ*^2^ (2) = 51.64	<0.001
School education	416 (41.5)	197 (48.6)			87 (53.4)		
Tertiary education	546 (54.5)	143 (35.3)		<0.001	51 (31.3)		<0.001
Years in education	14.69 (4.19)	12.94 (4.12)	*Z* = 7.07		12.58 (3.84)	*Z* = 5.92	
Social functioning							
Employment status			*Χ*^2^ (1) = 25.26	<0.001	79 (58.5)	*Χ*^2^ (1) = 0.48	0.487
Employed	615 (61.6)	141 (45.5)			56 (41.5)		
Unemployed	383 (38.4)	169 (54.5)					0.001
Marital status			*Χ*^2^ (1) = 126.23	<0.001		*Χ*^2^ (1) = 11.42	
Steady relationship	626 (62.4)	105 (28.3)			74 (48.1)		
No relationship	378 (37.7)	266 (71.7)			80 (52)		0.001
Living arrangements			*Χ*^2^ (1) = 96.98	<0.001		*Χ*^2^ (1) = 11.85	
Independent living	683 (68.5)	119 (37.5)			73 (53.7)		
No independent living	314 (31.5)	198 (62.5)			63 (46.3)		

s.d., standard deviation; df, degrees of freedom.

### Measures

#### Socio-demographics

Socio-demographic data were collected using the Medical Research Council (MRC) Socio-demographic Schedule modified version (Mallett, Leff, Bhugra, Pang, & Zhao, 2002), and supplemented by clinical records, with additional information on educational attainment and social functioning measured through employment, marital and living status.

#### Diagnosis

We used DSM-IV diagnosis(American Psychiatric Association, 1994) from interviews and mental health records utilising the Operational Criteria Checklist (OPCRIT) at baseline (McGuffin, Farmer, & Harvey, 1991) by centrally trained investigators, whose reliability was assessed throughout the study (*κ* = 0.7). These diagnoses were grouped into SSD group (codes 295.1–295.9 and 297.1–298.9) or AP group (patients diagnosed with codes 296–296.9), which was later stratified into BD (codes 296.0–296.06 and 296.4–296.89) and MDD with psychotic features (MDD-P, codes 296.2–296.36). For those subjects with missing information for DSM-IV output from OPCRIT, we reconverted ICD-10 diagnosis (*n* = 5) into DSM-IV codes; leaving eventually diagnostic data for 12 cases missing. Those who did not meet criteria from OPCRIT (i.e. undefined diagnosis) were not grouped into either of the groups (*n* = 52) and were excluded from further analyses.

### Genotyping and PRS building

All participants were invited to provide a genetic sample. DNA from blood tests or saliva samples was obtained from the majority of participants at baseline (73.6% of cases and 78.5% of controls), with no sociodemographic differences observed with those without genetic data except for minor age differences (please refer to the online Supplementary section 1.7). All DNA data collected were genotyped at the Cardiff University Institute of Psychological Medicine and Clinical Neurology, using a custom Illumina HumanCoreExome-24 BeadChip genotyping array covering 570 038 genetic variants; and quality control was performed locally (details provided in online Supplementary Material).

In order to control for population stratification, a Principal Component Analysis generating 10 principal components (PC) was run on pruned variants. After quality control of genetic and clinical data, and selection of individuals of European ancestry (details provided in online Supplementary Material), the genetic analyses included 573 cases (409 SSD, 74 BD and 90 MDD-P patients) and 1005 controls.

The measure of the aggregate genetic load is based on a PRS, which is an individual quantitative risk factor calculated from the weighted summation of the odds ratios of carried risk alleles taken from a discovery sample. It is represented by the following equation (Evans, Visscher, & Wray, 2009):
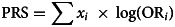
where *x* is the number of risk alleles of each included variant (*i*) and OR the respective odds ratio. To build the PRSs, results from the latest available GWAS which did not include the current EU-GEI sample, were used as discovery samples. In the case of SZ and BD, these were derived from the last mega-analyses of the PGC (Ripke et al., 2014; Stahl et al., 2019). Depression PRS was built from a GWAS combining PGC, 23andMe and UK Biobank samples (Howard et al., 2019; Ripke et al., 2014; Stahl et al., 2019). Finally, we further included PRS for IQ based on a large GWAS (Savage et al., 2018). All PRS were built using PRSice software (Choi & O'Reilly, 2019) at 10 different *p* value thresholds, and the selected *p* value threshold of 0.05 for SNP inclusion was chosen across the phenotypes on the basis of the published literature explaining the most variance in case–control analysis (Howard et al., 2019; Savage et al., 2018; Stahl et al., 2019; Wray et al., 2018). Each PRS was standardised to a mean of zero and a standard deviation of 1 (Lewis & Vassos, 2017). Variance explained in our sample at the different *p*-value thresholds are provided in online Supplementary Material (eFig. 1).

### Statistics

#### Descriptive statistics

Normality of all sociodemographic variables was assessed computing the Shapiro–Wilk normality test. The comparisons between cases and controls and between AP and SSD cases were made using chi-square, *t* test or Wilcoxon–Mann–Whitney tests when appropriate. Effect sizes were calculated for all the statistical tests using Cohen's *d* for *t* test and Cramer's *V* (*Φ_c_*) for chi-square. When Mann–Whitney test was used, effect sizes were calculated from *z* values.

#### Association analyses

We first analysed PRSs association with broad clinical groups (SSD, AP) by comparing them in cases only and also each group with controls; and in a second step, we measured the discrimination ability of PRSs between the two AP categories (BD and MDD-P) against SSD as a reference group, and then between each of the two. For this, we built a series of multinomial or simple logistic regression models in which we included the three disorder PRSs (PRS-SZ, PRS-BD, PRS-D) plus PRS-IQ as independent variables while controlling for population stratification using as confounders the first 10 PC and each sample site. Due to the inclusion of the four PRSs in the models, we adjusted the significance level as per Bonferroni's correction (Bland & Altman, 1995) dividing 0.05 by four, with a newly established significance level at *p* < 0.0125. Results will be presented in OR, 95% confidence intervals (CIs) and *p* value. Sensitivity post-hoc analyses controlling for 20 PCs did not significantly alter the results. We conducted power calculation analyses utilising the R-package AVENGEME (Dudbridge, 2013), which allows power calculation for PRS analyses. We calculated the required SNP-h^2^ or fix covariance in our target sample to obtain 80% of power on each regression model and per each PRS (SZ, BD and D).

As a secondary analysis, we explored the goodness of fit of data of the joint use of PRSs. We built a series of logistic regression models to test discriminability between AP and SSD in which, starting with a baseline model including PRS-SZ and 10PCs and sites as covariates, we sequentially added another PRS at a time, in order to identify those PRS adding significant value to the discriminability between the clinical groups by comparing models through likelihood ratio test (see online Supplementary Material for more details).

## Results

### Socio-demographics

Socio-demographics of the case–control sample are shown in Table 1, comparing SSD (*n* = 409) and AP (*n* = 164) with controls (*n* = 1005) separately. Compared with controls, patients were younger (mean age of 31.6, s.d. = 10.91 and 32.84, s.d. = 11.56 in SSD and AP respectively; 36.9, s.d. = 13 in controls); and a greater proportion of patients with SSD were men (68% *v*. 47%). Both SSD and AP were less likely to have received tertiary education and consequently reported fewer total years of education than controls (around over 12.5 years in cases and around 14.7 years for controls). Generally, cases were more likely not to be in a relationship and not to live independently. More SSD patients were unemployed, but no differences between AP and controls were found. Sociodemographic differences between clinical groups are provided in online Supplementary Material (eTable 3).

### PRS distribution in different clinical subgroups

**Fig. 1.**
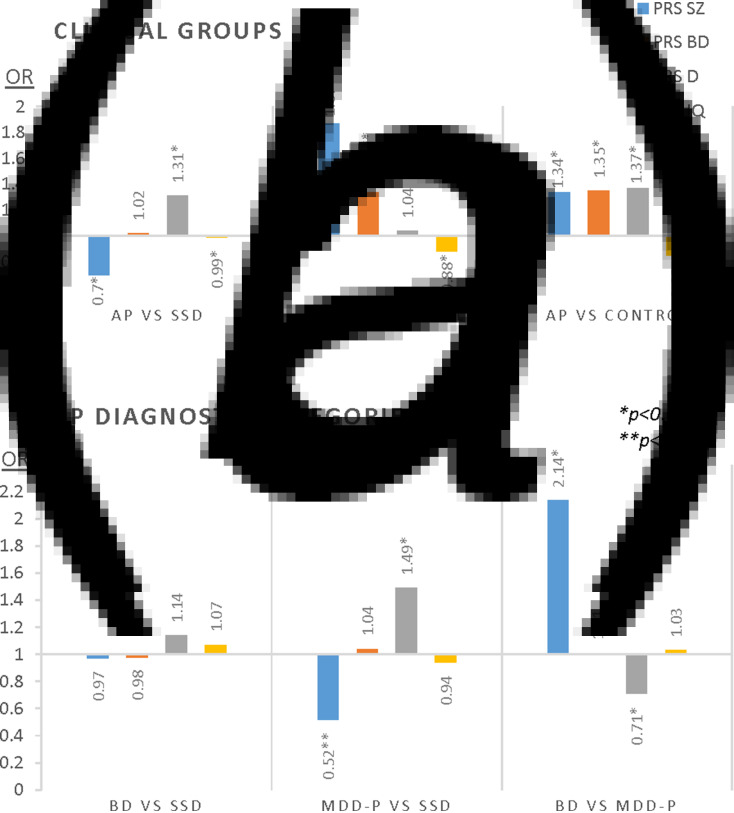
PRS performance for identifying clinical subgroups and categories based on DSM4 OPCRIT. Results in OR (*odds ratio*) based on multivariate models with all PRSs alongside 10PCs and sites as covariates. SZ, schizophrenia; BD, bipolar disorder; D, depression; IQ, intelligence quotient; SSD, schizophrenia-spectrum disorder (*n* = 409); AP, affective psychosis (*n* = 164); BD, bipolar disorder (*n* = 74); MDD-P, psychotic depression (*n* = 90). *p < 0.0125, **p < 0.001.

In the direct comparison between AP and SSD, both PRS-SZ and PRS-D were significantly associated with these diagnoses but in opposite directions (Fig. 1*a*). Whereas PRS-D (OR = 1.31, 95% CI 1.06–1.61, *p* = 0.011) was associated with increased risk of AP compared with SSD, the opposite was observed for PRS-SZ (OR = 0.7, 95% CI 0.54–0.92, *p* = 0.010). Hence, individuals with high PRS-SZ and low PRS-D have more chances of receiving a diagnosis of SSD, while low PRS-SZ and high PRS-D increases the chances of AP.

Regarding case–control comparisons with clinical subgroups, the first multinomial logistic regression showed that higher scores on both PRS-SZ and PRS-BD were associated with SSD (OR = 1.87, 95% CI 1.57–2.2, *p* < 0.001 and OR = 1.34, 95% CI 1.15–1.57, *p* < 0.001 respectively), whereas positive associations with AP were found for PRS-BD and PRS-D (OR = 1.35, 95% CI 1.09–1.67, *p* = 0.006 and OR = 1.37, 95% CI 1.14–1.64, *p* = 0.001 respectively). These effects are shown in Fig. 1*a* with additional details given in online Supplementary Material (eTable 4 and eFig. 2).

### PRS distribution between diagnostic categories within psychosis

In the second multinomial logistic regression, we tested whether PRSs could differentiate the two diagnostic categories included in AP (BD and MDD-P) from the broad group of SSD. As shown in Fig. 1*b*, no PRS was able to distinguish BD when compared with SSD. Nonetheless, the patterns for SSD and MDD-P diagnoses followed those observed above for SSD and broader AP comparisons. Thus, SSD and MDD-P diagnoses were differentiated by both PRS-SZ (OR = 0.52, 95% CI 0.37–0.74, *p* = 0.011) and PRS-D (OR = 1.49, 95% CI 1.14–1.94, *p* = 0.003) in the opposite direction. Further details are given in the online Supplementary Material (eTable 5).

When running simple logistic regression for discriminability between BD and MDD-P, only PRS-SZ could discriminate people diagnosed with BD from those diagnosed with MDD-P (OR = 2.14, 95% CI 1.23–3.74, *p* = 0.007) showing a positive association with the former.

### Fitting the model optimising PRS for SSD and AP discrimination

**Fig. 2.**
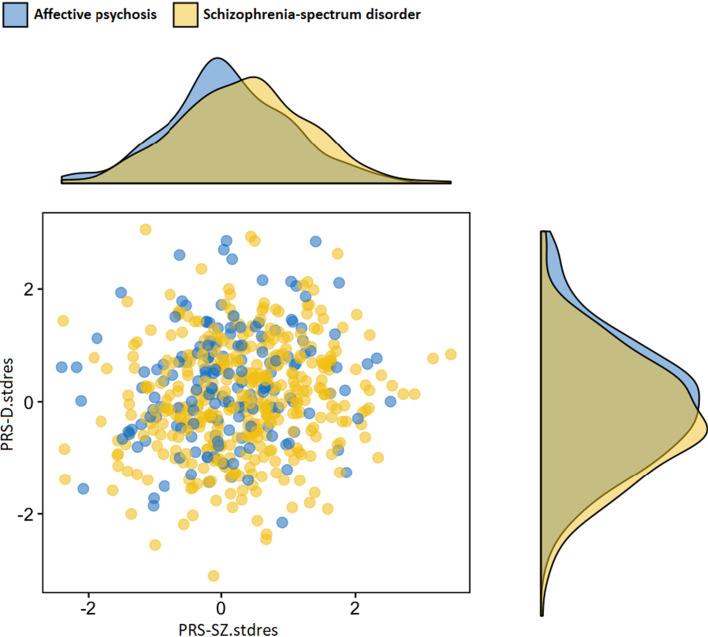
PRS-SZ and PRS-D distribution in cases with SSD and AP diagnosis. Scatterplot and density distributions of PRS-SZ and PRS-D in AP and SSD. Polygenic scores presented as z-score after adjustment for principal components and sites. Higher PRS-SZ increases the chances of SSD, while higher PRS-D increases the chances on affective psychosis.

In order to test which combination of PRSs better-differentiated SSD and AP as our main outcome, we built a series of regression models starting with a baseline model including PRS-SZ with covariates and sequentially adding the other three PRSs variables, once at a time. The best-fitting data as per likelihood ratio test was by adding PRS-D to the model (Δχ^2^(1) = 6.74, *p* = 0.0094) when compared with a model using only PRS-SZ. No further addition of PRS-BD or PRS-IQ improved the discrimination between clinical categories. Further details are provided in online Supplementary Material (eFig. 4). Based on these results, we plotted the distribution of standardised residuals of PRS-SZ and PRS-D, adjusting for 10 PCs, across the subgroups of SSD and AP (Fig. 2).

## Discussion

To the best of our knowledge, this is the largest multisite international case–control study to examine joint polygenic associations with specific diagnostic categories in FEP patients. Our study provides evidence to support an inverse gradient of PRS-SZ and PRS-D across diagnostic categories in the psychosis spectrum. Results also show a discriminability potential to distinguish the SSD from AP, especially from psychotic depression. No PRS was able to distinguish BD from SSD in this sample, while PRS-SZ was the only predictor that distinguished BD from psychotic depression (MDD-P). Moreover, we found that combining PRS for different disorders improves the prediction model for psychosis-related phenotypes.

### Interpretation of findings and comparison with other studies

The observed PRS-SZ associations which followed a gradient from SSD to AP categories (SSD > BD > MDD-P), are in line with the notion of a psychosis continuum across psychosis diagnostic categories and the observed genetic overlap between disorders (Cardno & Owen, 2014). Other studies have previously shown a similar PRS-SZ gradient (SZ > BD type I > BD type II) (Allardyce et al., 2017; Charney et al., 2017). However, PRS-SZ could not differentiate MDD-P from controls in our study. In a recent study, PRS-SZ seemed to be specially associated with those presenting psychotic features in the mania phase when compared with the depressive pole (Markota et al., 2018), which could explain our lack of association with MDD-P.

Previous research showed evidence of PRS for major depression (MDD) discriminated cases with depression from controls (Wray et al., 2018). Moreover, PRS for MDD failed to identify diagnostic subtypes in some case-only comparisons in BD (Charney et al., 2017), but seemed to be significantly associated with schizoaffective disorder depressed subtype when compared with SZ cases (Dennison et al., 2020). In our study, PRS-D differentiated psychotic depression from both controls and SSD, showing similar effect sizes as PRS-SZ in opposite direction. The discriminability potential of PRS-D in our sample may be due to the increased variance explained when selecting more severe patients with MDD (Verduijn et al., 2017) – only with psychotic features in our case -; the use of more powerful PRS-D built from PGC, UK Biobank, and 23andMe data (Howard et al., 2019); or that psychotic depression may be phenomenologically different to MDD without psychosis.

In relation to our main aim (i.e. whether we could use PRSs in order to distinguish between affective *v*. schizophrenia spectrum disorder subgroups), both PRS-SZ and PRS-D differentiated global AP from SSD, and psychotic depression from SSD. Nonetheless, when trying to differentiate BD from SSD, all PRSs failed to differentiate between them. This could be partly an artefact of the observed diagnostic instability in FEP; or be due to the fact that PRS-BD and PRS-D were underpowered for such analyses (more details in online Supplementary Material); but it is also plausible that this reflects the large genetic correlation between the two disorders, that may only be present to a lesser extent in depressive patients with psychotic features. Indeed, with over 80% power, PRS-SZ was able to distinguish BD from MDD-P, supporting the notion of lower common genetic liability for SZ in those suffering with psychotic depression than in those with BD, in line with the literature (Cross-Disorder Group of the Psychiatric Genomics Consortium et al., 2013).

**Fig. 3.**
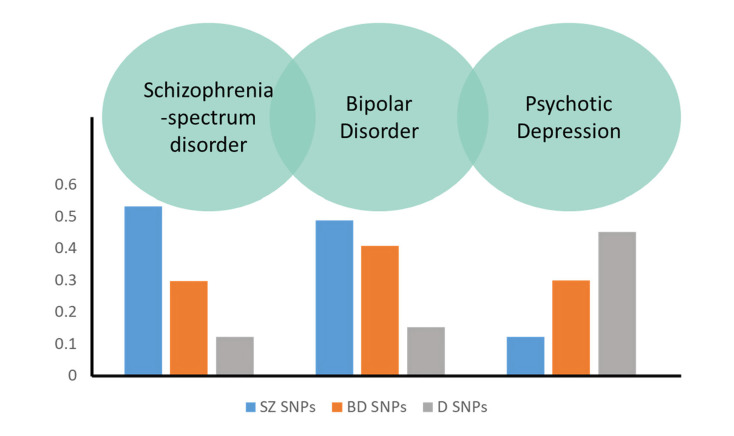
Visual representation of PRSs distribution across diagnosis categories. Conceptual multidimensional distribution of SNPs for Schizophrenia, bipolar disorder and depression across clinical groups. Based on mean case–control differences, using control as a reference of Standardised Residuals of PRS for SZ, BD and D adjusted by site and 10 principal components.

Our results are in line with a recently observed differentiation between psychotic disorders by using an aggregated genetic score based on family correlation (Kendler, Ohlsson, Sundquist, & Sundquist, 2021) and shed new light on the existence of yet unclear and blurred genetic boundaries between current diagnosis categories in their psychotic manifestation. Beyond the evidence of a gradient for risk of psychosis associated with PRS-SZ from SSD to the AP group, we could also observe an inverse gradient in the case of PRS-D. This allows the conceptualisation of a model in which the genetic vulnerability of psychotic disorders is distributed across a multidimensional continuum with SSD at one end, BD in the middle and MDD-P at the other extreme (Fig. 3). Among these groups, only the categories in the extremes were able to be differentiated by current polygenic scores. Further studies with larger samples or when the predictive power by PRSs increase, will allow further discrimination between categories, for example between SZ and BD or between BD and psychotic depression.

We failed to observe differences in PRS-IQ distribution, with effect sizes almost identical across clinical groups. Among AP, BD has been more widely compared with SZ as the paradigm disorder within SSD. We know from previous studies that patients with BD tend to present less cognitive impairment than those with SZ (Demjaha et al., 2017; Murray et al., 2004), but this difference seems to be less clear between SZ and BD patients with a history of psychotic symptoms (Hill, Harris, Herbener, Pavuluri, & Sweeney, 2007). Indeed, and in line with this, PRS-IQ showed no statistically significant differences within the case-only comparisons. However, the lack of discriminability potential of PRS-IQ would also be expected under the consideration that some cognitive changes are due to factors associated with the prodromal phase, the onset of the disorder or its treatment, rather than purely being neurodevelopmental, which is yet to be established.

These results should be interpreted in the context of some limitations. First, the number of patients with psychotic depression and BD was relatively small which could have led to low power in analyses comparing these groups and possibly contributing to the lack of association between those categories and most PRS variables. Furthermore, comparisons between models are also limited by the different discriminative power of each PRS (PRS-SZ is currently more powerful than PRS-BD and PRS-D). Indeed, *post-hoc* power calculations of the employed PRS suggest over 80% power only for PRS-SZ (more detail information in online Supplementary Material). These prediction models are expected to improve as bigger discovery samples are available for the affective psychotic categories and as we increase the size of our training sample. With FEP samples there are two main limitations to consider. One relates with the previously noted lower liability explained by PRS in incident samples (Meier et al., 2016), suggesting that part of the captured effect of SNPs is on the deteriorative course of illness, which may have implied type II error in our sample based on the FEP. The second limitation to consider refers to the changeability of diagnoses. As shown in some studies, shifts in diagnoses occur with a predominant direction from AP to SSD in a frequency of around 14–29% after 2 years (Schwartz et al., 2000; Veen et al., 2004). Moreover, it should be noted that all of our patients presented with psychosis, which could have enhanced the observed genetic overlap and prevented finding more clear differences between groups, and which make these results not generalisable to those BD or MDD without psychosis. Finally, all analyses were performed in the people of European ancestry population, which limits the generalisability of the findings in other populations. However, the fact that this is a multicentre well-characterised sample of FEP, allows it to have generalisability within Caucasian European populations.

Overall, this study provides support for the presence of a genetic psychosis continuum (shown by the ability of PRS-SZ to differentiate most case groups from controls following a gradient across categories). Nonetheless, we also observed genetic differences between clinical categories, with schizophrenia spectrum disorders at one end and psychotic depression at the other when looking at genetic loading for SZ and D. This study also shows that combining PRSs for different disorders in a prediction model of psychosis related phenotypes improve our prediction models while contributing to our understanding of the biological underpinnings of these phenotypes. Despite not yet clinically applicable at an individual level, this study points towards the potential usefulness as a research tool in specific populations such as high-risk or early psychosis phases, where it may help to suggest different therapeutic approaches (i.e antidepressant *v.* antipsychotic) or to anticipate prognosis. However, further work is needed to explore if PRS have synergistic effects with environmental exposures before combining all the risk factors into a single prediction model.

## Supplementary material

For supplementary material accompanying this paper visit https://doi.org/10.1017/S0033291721005456. click here to view supplementary material
